# Investigating the physical and chemical factors affecting the microbial status of water in the water distribution network of Babol City with an emphasis on the HPC index

**DOI:** 10.1371/journal.pone.0324186

**Published:** 2025-06-12

**Authors:** Hossein Faraji, Hadi Rahimzadeh, Mehdi Salari, Zahra Aghalari, Fatemeh Asgharzadeh, Somayyeh Jafarian

**Affiliations:** 1 Department of Environmental Health Engineering, School of Public Health and Allied Medical Sciences, Iranshahr University of Medical Sciences, Iranshahr, Iran; 2 Department of Environmental Health Engineering, Golestan University of Medical Sciences, Gorgan, Golestan, Iran; 3 Student Research Committee, Sabzevar University of Medical Sciences, Sabzevar, Iran; 4 Department of Environmental Health Engineering, School of Health, Sabzevar University of Medical Sciences, Sabzevar, Iran; 5 Environmental Health Engineering, Babol University of Medical Sciences, Babol, Iran; 6 PhD in Environmental Health Engineering, Department of Environmental Health Engineering, Babol University of Medical Sciences, Babol, Iran; 7 Environmental Health Engineering, Vice-Chancellor of Health, Babol University of Medical Sciences, Babol, Iran; 8 Tropical and Communicable Diseases Research Center, Iranshahr University of Medical Sciences, Iranshahr, Iran; Gujarat Institute of Desert Ecology, INDIA

## Abstract

**Background and purpose:**

Safe and hygienic drinking water is crucial for public health. Secondary bacterial contamination, often caused by improper transport and distribution conditions, poses a significant threat. This study aimed to investigate the physical and chemical factors influencing the microbial status of drinking water in Babol’s distribution network, focusing on the heterotrophic plate count (HPC) index.

**Methods:**

Thirty-two water samples were collected randomly from Babol’s rural areas and analyzed for HPC, residual free chlorine, turbidity, pH, and temperature. HPC was measured using the plate spreading technique on R2A agar. Descriptive statistics and regression analysis were employed to analyze the data.

**Findings:**

Heterotrophic bacteria were detected in 61.4% of the samples, with 9.6% exceeding 500 CFU/ml. The average HPC was 140 CFU/ml, while residual free chlorine averaged 0.33 mg/l. In 21.4% of samples, residual chlorine was below 0.2 mg/l, and 19% had no detectable chlorine. Turbidity ranged from 0.05 to 3 NTU. Statistical analysis revealed significant positive correlations between HPC and pH, MPN, turbidity, and temperature (α < 0.05).

**Conclusion:**

Regular HPC monitoring in Babol’s drinking water distribution network is essential to identify contaminated areas and ensure adequate residual free chlorine levels (0.2–0.8 mg/l) for maintaining bacteriological water quality.

## Introduction

One of the causes of infectious diseases is microbial contamination of drinking water [1]. Controlling water-borne diseases is a primary goal of water supply projects; therefore, the WHO has prioritized microbial quality control in drinking water supply [2]. According to the WHO report, one in ten diseases worldwide is caused by unhealthy water, and 6% of deaths globally are attributed to unhealthy water [3]. Water microbial contamination occurs at various points in water supply projects, including source waters and the distribution network [4]. Surveys have shown that the drinking water distribution network can be a source of water quality degradation for several reasons [5].

While the most common indicator of microbial quality in drinking water is the measurement of coliform and thermogenic coliform bacteria, Heterotrophic Plate Count (HPC) bacteria are also recommended for evaluating the filtration system and distribution network in many cases [6,7]. Heterotrophic bacteria encompass a wide range of bacteria found in water, dust particles, soil, etc. [8]. Except for Bacillus and Micrococcus, most heterotrophic bacteria are gram-negative and include Proteus, Enterobacter, Aeromonas, Citrobacter, Pseudomonas, Klebsiella, Flavobacterium, Serratia, Moraxella, and Alkaligenes [9]. Pseudomonas species are known to cause skin and lung infections, and Aeromonas species can cause opportunistic gastroenteritis [10].

High populations of heterotrophic bacteria in water can affect the health of individuals with immune system defects, children, infants, the elderly, and people who have suffered severe burns [11]. The HPC method is considered an effective indicator in water purification, and many scientific sources emphasize its monitoring of drinking water to ensure consumer health. This method measures the number of living heterotrophic bacteria in water [12,13]. The American Environmental Protection Agency has set the maximum allowable number of heterotrophic bacteria in the drinking water distribution network at 500 CFU/ml [14].

In drinking water, the number of HPC bacteria can range from less than one CFU to more than 104 CFU per milliliter and is primarily influenced by temperature, residual chlorine, and absorbable organic matter [6,15]. A high number of HPC bacteria in drinking water indicates contamination, although a sudden increase in colony count may signal new contamination in water sources [16]. Studies have shown contamination of drinking water samples with HPC in various regions, such as 3.5% of samples in Germany [17] and 45% of samples related to underground water in Rohri city, Pakistan [18].

The presence of heterotrophic bacteria in high densities can make it difficult to accurately determine fecal contamination and pathogenicity [19]. High densities of heterotrophic organisms, exceeding 1000 CFU/ml in water samples, can reduce the sensitivity of the multi-tube test and membrane filter method [20]. Pathogenic bacteria such as Legionella and Aeromonas may be present in the water, and the absence of coliform does not guarantee the absence of these microorganisms. Some studies have also demonstrated the interference of high heterotrophic populations in coliform experiments using lactose culture [21].

High populations of heterotrophic bacteria in the drinking water distribution network, in addition to aesthetic issues (taste, smell, color, and biofilm production), are of economic concern due to corrosion and sediment production. Some heterotrophic bacteria are considered pathogenic, while others are opportunistic [22]. HPC control, in conjunction with other information, can be used to validate and improve the efficiency of water treatment processes such as filtration and disinfection [13]. In the distribution network, HPC can also provide insights into the condition of the water supply network, including the presence of organic matter, reduced disinfectant residuals, nutrient availability, lag time, and pressure drops [23].

Various techniques, such as plate pouring, plate diffusion, and membrane filtration, have been used to identify and measure heterotrophic bacteria in water [24]. Cultivation method, culture medium composition, culture temperature, and incubation time are important parameters for culturing these bacteria. The HPC method is typically evaluated in relation to variables such as turbidity, residual chlorine, pH, and temperature in water [25,26]. As the HPC method is not a standard bacteriological quality control test for drinking water, no data has been published for the city of Babol in this area. This research was conducted to investigate the physical and chemical factors affecting the microbial status of water in the water distribution network of Babol City with an emphasis on the HPC index.

## Materials and methods

### Study area

Babol Plain is located in central Mazandaran province, with geographical coordinates ranging from 38° 52’ to 25° 52’‘ longitude and 37° 36’‘ to 54° 35’‘ latitude. Babol Plain, covering an area of 1578 m², has over 510 villages with a rural population of 230,973. The drinking water supply sources for Babol villages consist of 35 wells (single village and complex) and 3 springs.

### Study design

This study was descriptive and cross-sectional, conducted from 2021 to 2022, examining 38 water well sources and analyzing HPC parameters, residual free chlorine, MPN, pH, temperature, and turbidity. Permission was obtained from the Research and Technology Department of Babol University of Medical Sciences to conduct this study, and this study was registered and approved by the Ethics Committee (IR.MUBABOL.HRI.REC.1401.018). Research ethics were observed to conduct this research.

### Sample collection

Sterile glass containers containing 3% sodium thiosulfate were used to collect water samples, which were then transported to the laboratory under refrigeration. Temperature and residual free chlorine were measured at the sampling site and analyzed according to standard methods provided by APHA for water and sewage testing [27].

### Heterotrophic plate count

The plate pouring method and R2A agar culture medium were used for heterotrophic plate count. All samples were incubated for 72 hours at 35°C.

### Data analysis

**Fig 1:**
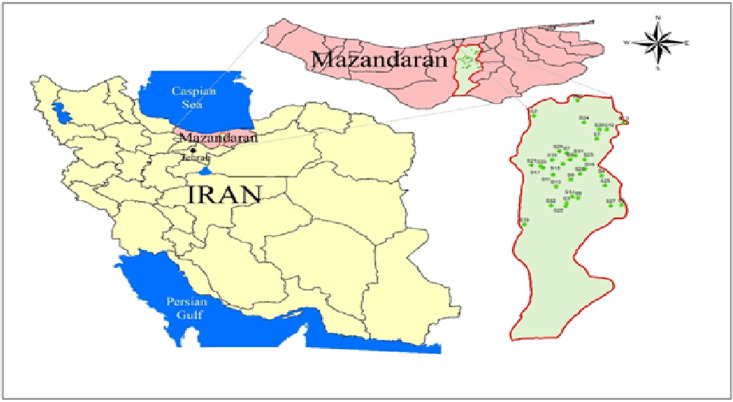
Map of selected groundwater source’s location in Babol.

Descriptive statistics and correlation analysis between variables were performed using the regression statistical test method with SPSS-61 software. The location of the wells was recorded using GPS. Fig 1 shows the location of the selected groundwater source in Babylon.

Coliform bacteria were studied using Lactose Broth and Brilliant Green Lactose Broth (BGB), while heterotrophic bacteria were studied using R2A culture medium. The MPN test was conducted as outlined in the standard book [28]. A lactose broth culture medium was used in the possible stage of the multi-tube fermentation experiment.

For drinking water, ten tubes containing 10 ml samples were prepared. After 24 hours of incubation at 35°C, the tubes were examined for microbial growth and gas production. Negative results after 48 hours indicated the absence of coliform bacteria. Positive tubes were transferred to the confirmation stage.

In the confirmation stage, fermentation tubes of Brilliant Green Lactose Broth (BGB) were used. Tubes with gas and acid production were gently shaken to distribute the organisms. Using a sterile loop, one or more complete loops were transferred from the fermentation tube of the first stage to the fermentation tube containing Brilliant Green Lactose Broth in the confirmation stage. The tubes were incubated at 35°C. Gas formation in Durham tubes within 48 hours indicated a positive result.

The HPC test was performed using the Spread Plate Count method, with incubation at 35°C for 72 hours [29]. Biochemical tests and Gram staining were used to identify isolated bacterial strains [30].

Temperature was measured with a portable thermometer (Metrohm), free residual chlorine was measured with a DPD chlorometer kit, and pH was determined using a digital pH meter model 827 (Metrohm). Turbidity was measured using a turbidity meter model A2100 (HACH) NTU.

## Findings

Table 1 summarizes the data results using descriptive statistics parameters (range, mean, and standard deviation). In addition, the results of paired t-test analysis are presented in the table. The results of the experiments showed that the average, maximum and minimum pH in summer were 7.5 ± 0.38, 9 and 6.45, and in winter they were 6.96 ± 0.33, 7.8 and 6.5, respectively. the average, maximum and minimum RCvalues were 0.2 ± 0.21, 0.9, and 0 mg/l in summer and 0.2 ± 0.2, 0.9, and 0 mg/l in winter. the average, maximum and minimum HPC were 48.5 ± 233, 900, and 0 (CFU/ml)in summer and 51.5 ± 221, 905, and 0 (CFU/ml)in winter. Table 1 shows that there is no significant difference in pH and RC values between the two seasons. In contrast, the parameters turbidity, temperature (T), probable number (MPN), and heterotrophic plate number (HPC) show significant differences across seasons. Given the presence of these samples in the two seasons, the t-test was used for analysis. The results are presented in Table 1. The concentrations of HPC and MPN in urban drinking water were higher during summer than in winter.

**Table 1:** Results of statistical (descriptive) analysis of data in positive samples collected from drinking water sources.

Statistical analysis (descriptive)	Season	pH	RC mg/l	Turbidity	T	MPN	HPC (CFU/ml)
Average ±	Summer	7.5 ± 0.38	0.2 ± 0.21	0.5 ± 0.52	20 ± 1.34	0 ± 9.7	48.5 ± 233
	winter	6.96 ± 0.33	0.2 ± 0.2	0.7 ± 43	18.5 ± 1.32	0 ± 8.3	51.5 ± 221
Paired samples T-test		0.457	0.742	0.05>	0.05>	0.05>	0.05>
Minimum	Summer	6.45	0	0.06	16.9	0	0
	winter	6.5	0	0.26	16.8	0	0
Maximum	Summer	9	0.9	3	23.2	37.2	900
	winter	7.8	0.9	3.2	21.6	35.0	905

### Distribution map of rural water resources quality parameters in Babol

#### Chlorine residual distribution map in Babol rural aquifer.

**Fig 2:**
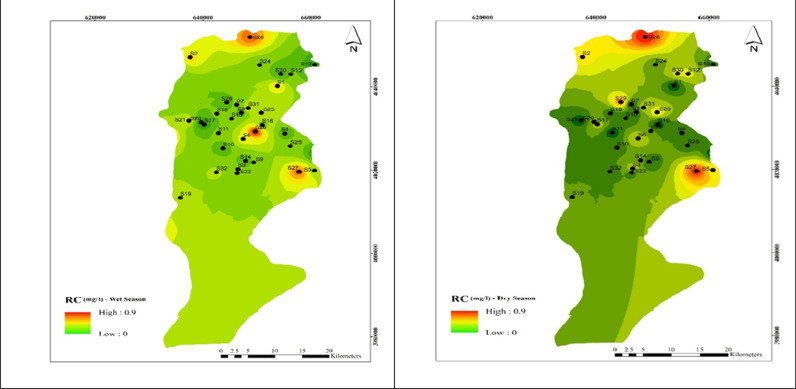
Spatial distribution of residual chlorine during (a) wet and (b) dry season samples.

As shown in Fig 2, the residual chlorine concentration in all 34 wells was below the Iranian national standard of 0.5–1 mg/l. The highest residual chlorine levels were found in wells 26 and 28, reaching 0.9 mg/l in the cold season and 0.9 mg/l in the dry season. Overall, the average residual chlorine in water sources throughout the year was 0.25 ± 0.23 mg/l, with no statistically significant difference between spring and summer α0.05.

**Fig 3:**
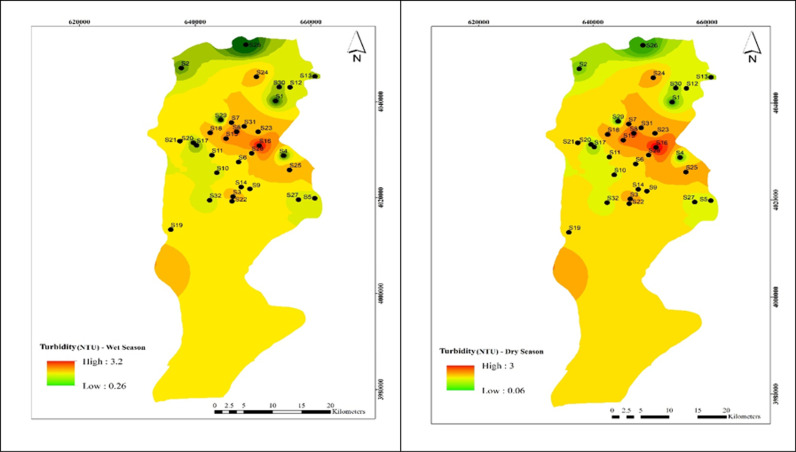
Spatial distribution of turbidity during (a) wet and (b) dry season samples.

As shown in Fig 3, the turbidity concentration in all 34 wells was below the Iranian national standard of 5 mg/l. The highest turbidity levels were found in well 16, reaching 3 mg/l in both the dry and cold seasons. Overall, the average water turbidity throughout the year was 0.75 ± 0.53 mg/l, with no statistically significant difference between spring and summer(α > 0.05).

**Fig 4:**
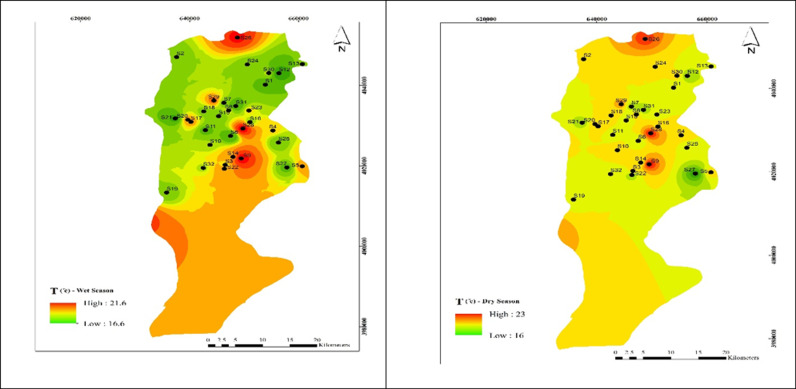
Spatial distribution of temperature during (a) wet and (b) dry season samples.

As shown in Fig 4, the highest summer and winter temperatures were recorded in wells 9, 26, and 28, reaching 23°C in summer and 21.4°C in winter. The average water temperature in the Babol Plain was 18.7 ± 1.3°C throughout the year, with a statistically significant difference between spring and summer (α < 0.05).

Therefore, the water temperature in the Babol Plain falls within the permissible range.

**Fig 5:**
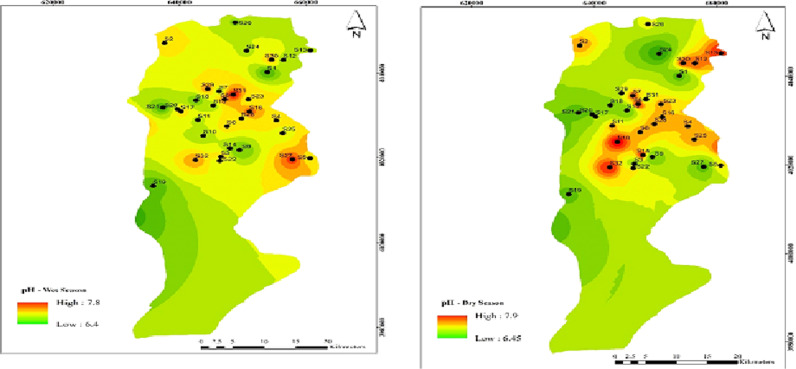
Spatial distribution of pH during (a) wet and (b) dry season samples.

As shown in Fig 5, the pH concentration in all 34 wells was within the Iranian national standard of 6.5–9. The highest pH was recorded in well 31 during the cold season at 7.8. The average pH in the Babol Plain was 7.19 ± 0.39 throughout the year, with no statistically significant difference between cold and dry seasons(α > 0.05).

**Fig 6:**
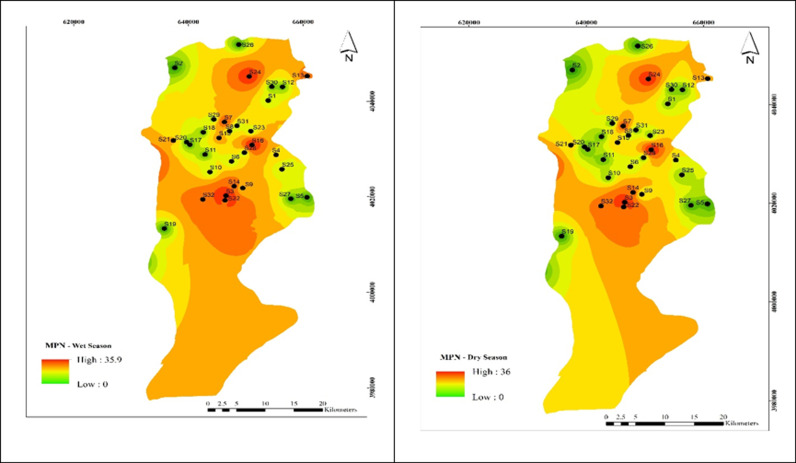
Spatial distribution of MPN during (a) wet and (b) dry season samples.

As shown in Fig 6, the MPN concentration in all 34 wells was 0–36 The highest MPN was recorded in well 3,13, 18, 22, 24 in the cold season with 35.6. The average MPN was recorded in well 3,13, 18, 22, 24 in the coldof the Babol Plain throughout the year was 36, which was not statistically significantly different between the cold and dry seasons(α > 0.05).

**Fig 7:**
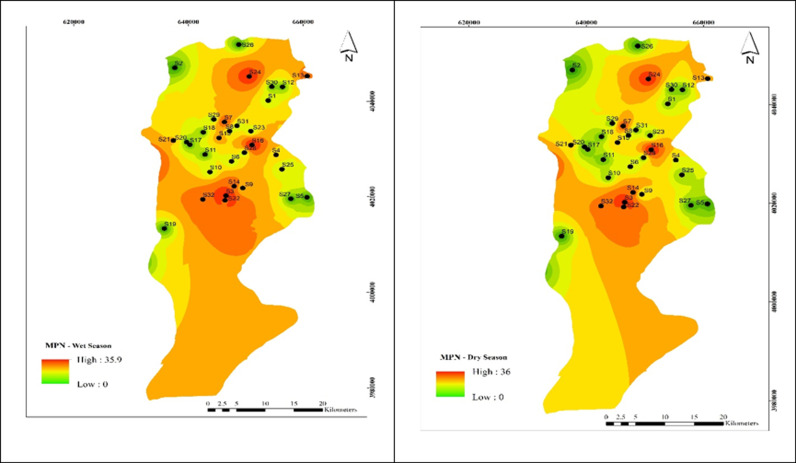
Spatial distribution of HPC during (a) wet and (b) dry season samples.

As shown in Fig 7, the HPC distribution map indicates the highest HPC levels in wells 16, 22, and 24, reaching 903 and 805 CFU/ml in the cold season and 900, 789, and 768 CFU/ml in the dry season. The average HPC in Babol Plain water sources throughout the year was 164.8 ± 232.4 CFU/ml, with no statistically significant difference between cold and dry seasons(α > 0.05)

Table 2 presents the Pearson correlation coefficients between HPC and various water quality parameters, including pH, chlorine, turbidity, MPN, and temperature.

**Table 2:** Pearson correlation coefficients between HPC and various water quality parameters.

Variable	RC	Turbidity	T	pH	MPN
HPC	−0.42	+0.54	−0.21	+0.25	+0.6

Numbers represent Pearson’s correlation coefficient (R).

The relationship is significant at the α = 0.05

The relationship is significant at the α =0.01

Statistical analysis (Pearson’s test) revealed significant correlations between HPC and several water quality parameters:

- **Inverse relationships:** HPC decreased with increasing residual chlorine (RC) (R = −0.42) and water temperature (R = −0.21).
- **Direct relationships:** HPC increased with increasing turbidity (R = 0.54), pH (R = 0.25), and MPN (R = 0.6).

## Discussion

Microorganisms can grow in water as layers (biofilms). They can also adhere to surfaces that have been in contact with water. Factors such as large changes in the concentration of free residual chlorine [31], especially its sudden decrease in drinking water, pipe breakage, etc., cause microorganisms to enter the water environment and, as a result, increase a wide range of HPC [32]. The results of the study showed that heterotrophic bacteria grew in most of the different wells in rural Babol.

According to Table 1, there is no significant difference in pH and RC values between the two seasons. In contrast, the parameters turbidity, temperature (T), probable number (MPN), and heterotrophic plate number (HPC) show significant differences across seasons.Due to the presence of these samples in two seasons, a t-test was employed for the analysis. The results are presented in Table 1. The higher levels of HPC and MPN in urban drinking water during the summer compared to winter can be attributed to several factors: Temperature Impact: Elevated temperatures in summer can enhance microbial activity. Microorganisms tend to grow more actively in warmer conditions. Reduced Precipitation: There may be less rainfall in summer, leading to the accumulation of pollutants in water sources [33].

Water Flow: Decreased water flow during summer can result in higher concentrations of microorganisms. Human Activities: Increased human activities such as tourism, agriculture, and industrial operations during summer can contribute to higher levels of water pollution [34].

Wastewater Discharge: There may be increased discharge of wastewater into water sources during the summer months. Inadequate Treatment: The higher demand for water in summer may place additional stress on water treatment systems, potentially compromising treatment quality [35].

These factors can collectively lead to increased levels of HPC and MPN in urban drinking water during the summer compared to winter

### Effect of residual free chlorine on HPC concentration

As shown in Table 1, the average residual chlorine (RC) in the wet and dry seasons was 0.2 ± 0.21 and 0.2 ± 0.2 mg/l, respectively. The national standard requires a minimum RC of 0.2 mg/l. The maximum and minimum chlorine levels measured in this study were 0.8 and 0.6 ppm, respectively.

Table 2 illustrates that residual chlorine has a significant inverse relationship with HPC bacteria count (α < 0.01). As residual chlorine increases, the number of bacterial colonies in water sources decreases, primarily due to the bacteria’s sensitivity to chlorine. The findings of Pindi et al. (2013) and Tavangar et al. (2014) align with this study [36,37]. A study by Kalantary et al. (2024) showed that residual chlorine reduced microbial contamination of pool water. Also, the use of clarifiers along with the concentration of free residual chlorine can help ensure the bacteriological quality of pool water, and maintaining the daily residual chlorine level of the pool can be effective in controlling heterotrophs in pools and aquatic recreational environments [38]. Dias et al. (2019) showed that disinfectant residues such as chlorine and hydraulic retention time are determining factors in drinking water quality [39].

### Effect of turbidity on HPC concentration

Turbidity, a measure of water transparency, can promote pathogen growth and proliferation while reducing the effectiveness of water disinfection [40]. The US-EPA recommends a maximum allowed turbidity of 5 NTU and a desirable value of 1 NTU [41]. However, the World Health Organization sets a lower standard of less than 1 NTU [42].As shown in Fig 4, the highest turbidity values were 2.48 NTU and 1.62 NTU in regions 3 and 9, respectively. The lowest turbidity level of 22 NTU was measured in the area. This value falls within Iran’s national standard of 5 NTU. The turbidity measured in the distribution network ranged from 0.18 to 1.05 NTU, remaining below the permissible limit.Pearson correlation analysis in Table 2 revealed a significant direct relationship between turbidity and HPC bacteria count (α < 0.01). Hass et al.’s (1983) study supports these findings [43]. Liu et al. (2016) showed that show that the presence of turbidity and suspended particles leads to an increase in the presence of bacteria in drinking water and reduces water quality [44].Table 2 further confirms the direct significant relationship between turbidity and HPC ray bacteria count (α < 0.01), aligning with the study of Gholami et al. (2022) [45].

### Effect of temperature on HPC concentration

While water temperature in drinking water distribution networks may not have significant health effects [46], the World Health Organization (WHO) recommends a maximum temperature of 25 °C for drinking water [47].As shown in Table 1 and Fig 5, there was a direct relationship between HPC and temperature, but it was not significant, aligning with the findings of studies conducted in Isfahan [41] and Semnan [48]. Water temperature in drinking water distribution networks does not have significant health effects and cannot be adjusted to public health standards. However, in general, it can be noted that water temperature can have effects on water purification, aquatic animal life, and the rate of chemical reactions, and from this perspective, water temperature is important.

### Effect of pH on HPC concentration

The average pH in the wet and dry seasons was 7.5 ± 0.38 and 6.96 ± 0.33, respectively, as shown in Table 1. The WHO standard recommends a pH range of 6.5 to 9.5, with an optimal range of 7 to 8.5. All drinking water wells in Babol fell within the natural water range according to WHO standards.

Fig 5 indicates that pH values were similar in both dry and wet seasons, with a minimum of 7.3 and a maximum of 7.7. Well 1 had the highest pH in both seasons. The groundwater in the study area is naturally alkaline.Pearson’s correlation analysis revealed a significant relationship between HPC and pH (α < 0.01). As pH increases, the potential count of coliform bacteria also increases. These findings are consistent with the study by Kulthanan et al. (2013) [49]. The study of the ionization reaction of hypochlorous acid shows that at low pH, chlorine is mostly in the form of hypochlorous acid and at high pH, chlorine is mainly in the form of hypochlorite ion in water. The effect of hypochlorous acid in disinfecting fecal bacteria and unicellular organisms is 100 times greater than that of hypochlorite ion. Therefore, with increasing pH, the disinfecting power of chlorine decreases significantly.This justifies the results of the present study that the amount of bacteria increases with increasing pH because the disinfecting power of chlorine decreases with increasing pH.

### Effect of Most Probable Number (MPN) on HPC concentration

Identifying indicator bacteria is a crucial method for evaluating the effectiveness of water disinfection methods [48]. The most important indicator bacteria include Escherichia coli, other thermophilic coliforms, and coliforms [50]. The MPN method is suitable for isolating and identifying these bacteria [51].As shown in Table 1 and Fig 6, the highest populations of heterotrophic bacteria were counted at 70 and 115 cfu/ml in regions 3 and 9, respectively. This was observed in only 8% of the regions. According to the recommended standard, the maximum allowed level is up to 500 cfu/ml. Amanidaz et al. (2015) reported that in 114 samples, heterotrophic bacteria exceeded 500 CFU/ml. Fecal coliform, total coliform, and streptococcus levels were 8, 32, and 20 CFU/100 ml, respectively. A significant relationship was found between heterotrophic bacteria, coliforms, and fecal streptococci [6].Table 2 demonstrates a significant direct relationship between HPC and the MPN method (α < 0.01). Nazimi et al. found that heterotrophic plates exceeded 500 in 8.19% of villages [52]. Masafari et al. observed heterotrophic bacteria in 50% of samples from different areas of the city, with counts exceeding 500 in 6 areas [53]. Kacar et al.‘s study (2017) showed a negative correlation between physicochemical parameters and fecal bacteria levels but found no significant correlation using the Pearson correlation test [54].In the present study, the highest HPC levels were found in regions 3, 6, and 9, with less than 3 colonies in other parts of the city, aligning with the findings of the two brothers and colleagues study [41]. Statistical analysis revealed significant relationships between the population of heterotrophic bacteria, turbidity, and residual free chlorine (α < 0.05). Masafari et al. also reported a significant relationship between HPC and residual chlorine (α < 0.01 and R = 0.6) [53].

## Conclusion

This study investigated the relationship between heterotrophic plate count (HPC) and physicochemical parameters in drinking water. The results indicate that residual free chlorine is a critical factor in controlling HPC levels. Higher residual chlorine concentrations are associated with lower HPC counts, suggesting the effectiveness of chlorine disinfection. Turbidity also plays a role, with increased turbidity leading to slightly higher HPC levels, especially when turbidity exceeds 1 NTU.Statistical analysis confirmed a significant direct relationship between HPC and turbidity at the α = 0.01 level. Both turbidity and pH had a positive correlation with HPC, while residual free chlorine and temperature had inverse correlations. However, the intensity of these relationships varied, with residual free chlorine and turbidity having the strongest impacts on HPC.Overall, this research highlights the importance of maintaining adequate residual chlorine levels and controlling turbidity to ensure safe and sanitary drinking water. By addressing these factors, water utilities can effectively reduce HPC and improve the quality of potable water. Knowing the quality status of surface and groundwater using water quality indicators makes it possible to adopt management strategies that will cause the least damage to this important and vital resource, while using the information obtained when necessary. Water resources management has different requirements and variables, each of which plays a determining role. One of the most important and fundamental of these variables is water resource monitoring. Water quality monitoring operations are the planned process of sampling, measuring, and recording or marking various water characteristics, often with the aim of assessing the suitability and compliance with the defined purpose or goals of water use. Monitoring operations, while providing the information needed to understand the current situation, the quality of water resources by determining quality fluctuations, provide a suitable basis for timely control measures and also determine the quality trend of water resources for the development of protection and exploitation programs.The limitations of the present study include the small sample size and the small sample size. The small sample size in Babol city was due to various reasons such as economic problems and insufficient budget, lack of sufficient laboratory equipment, and lack of sufficient manpower (environmental health experts) for sampling. It is hoped that these limitations will be overcome in future research.

## Supporting information

S1 Dataset Minimal dataset. (XLSX)

## Abbreviations

WHO: World Health Organization
HPC: Heterotrophic Plate Count
BGB: Brilliant Green Lactose Broth
pH: Potential of Hydrogen
MPN: Most Probable Number.
